# Update to: Two Randomized Phase 3 Studies of Aducanumab in Early Alzheimer's Disease

**DOI:** 10.14283/jpad.2024.147

**Published:** 2024-07-01

**Authors:** C. Rubel, T. Chen, Gersham Dent

**Affiliations:** 1 Biogen Inc, Cambridge, MA, USA

*Dear Editor*,

The article, “Two Randomized Phase 3 Studies of Aducanumab in Early Alzheimer's Disease,” authored by Haeberlein SB, et al, was originally published on March 18, 2022. This article reported plasma p-tau181 data from 870 and 945 patients in the EMERGE and ENGAGE studies, respectively. Since the publication of the original article, additional plasma p-tau181 data from EMERGE and ENGAGE have become available. Therefore, there are several updates that need to be made to the original publication:

• In the “Clinical assessments and biomarker substudies” section, “A total of 6684 plasma samples (n=3474 from EMERGE and n=3210 from ENGAGE) were analyzed” should be updated to “A total of 6940 plasma samples (n=3043 from EMERGE and n=3897 from ENGAGE) were analyzed.”
• In the “Efficacy” section, “Plasma p-tau was assayed in 870 and 945 patients in EMERGE and ENGAGE, respectively” should be updated to “Plasma p-tau was assayed in 885 and 953 patients in EMERGE and ENGAGE, respectively.”
• In the same section, “The difference in adjusted mean change from baseline between high-dose aducanumab and placebo was −0.667 (95% CI, −0.860 to −0.474; *P*<.0001) for EMERGE and −0.777 (95% CI, −0.931 to −0.623; *P*<.0001) for ENGAGE” should be updated to “The difference in adjusted mean change from baseline between high-dose aducanumab and placebo was −0.669 (95% CI, −0.858 to −0.479; *P*<.0001) for EMERGE and −0.769 (95% CI, −0.922 to −0.616; *P*<.0001) for ENGAGE.”
• With the additional plasma p-tau181 data from EMERGE and ENGAGE, Figures 2c and d have also been updated as indicated with red boxes. In the enclosed supplementary materials, Supplemental Data Table 1 and Supplemental Data Figures 4a and c have also been updated as indicated with red boxes.

**Figure 2:**
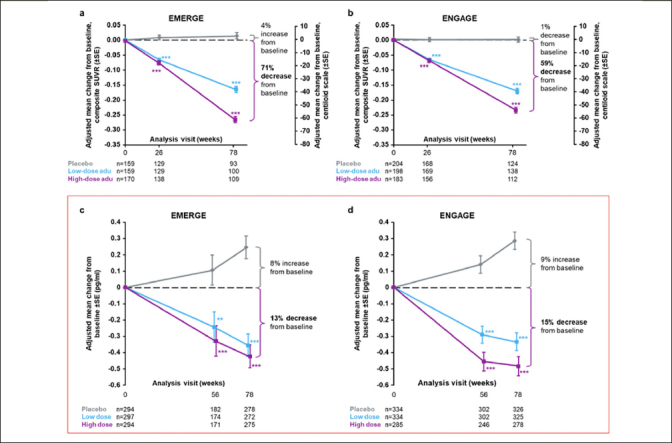
Longitudinal change from baseline in amyloid PET composite SUVR (and centiloid) assessed by ^18^F-forbetapir in the amyloid PET substudies of EMERGE (a) and ENGAGE (b) In panels (a) and (b), percentages from baseline are based on the centiloid scale. The composite SUVR was computed from the frontal, parietal, lateral temporal, sensorimotor, anterior, and posterior cingulate cortices and normalized using the cerebellum as the reference region. Longitudinal change from baseline in plasma p-tau181 levels in the plasma p-tau^181^ analysis populations from EMERGE (c) and ENGAGE (d); **P<.01. *** *P*<.001. Error bars denote SEs; adu, aducanumab; PET, positron emission tomography; SUVR, standardized uptake value ratio.

While we feel it is important to communicate updates to the data, it is important to note that these analyses with new sample sizes for each trial have not changed any of the original conclusions.

*Disclosures:* These studies were funded by Biogen. CR, TC, and GD are employees and shareholders of Biogen. Medical writing support, under direction of the authors, was provided by Nucleus Global and was funded by Biogen.

## Electronic Supplementary Material

Supplementary material, approximately 333 KB.

