# Supplementary material for: Update to: Two Randomized Phase 3 Studies of Aducanumab in Early Alzheimer's Disease
Source: J Prev Alzheimers Dis. 2024 Jul 1;11(4):1178–9. doi: 10.14283/jpad.2024.147 (PMC11266211; doi:10.14283/jpad.2024.147)
Supplement: Supplementary file 1 — Supplementary material, approximately 333 KB. [file mmc1.docx]

**Supplemental Data Table 1**

**
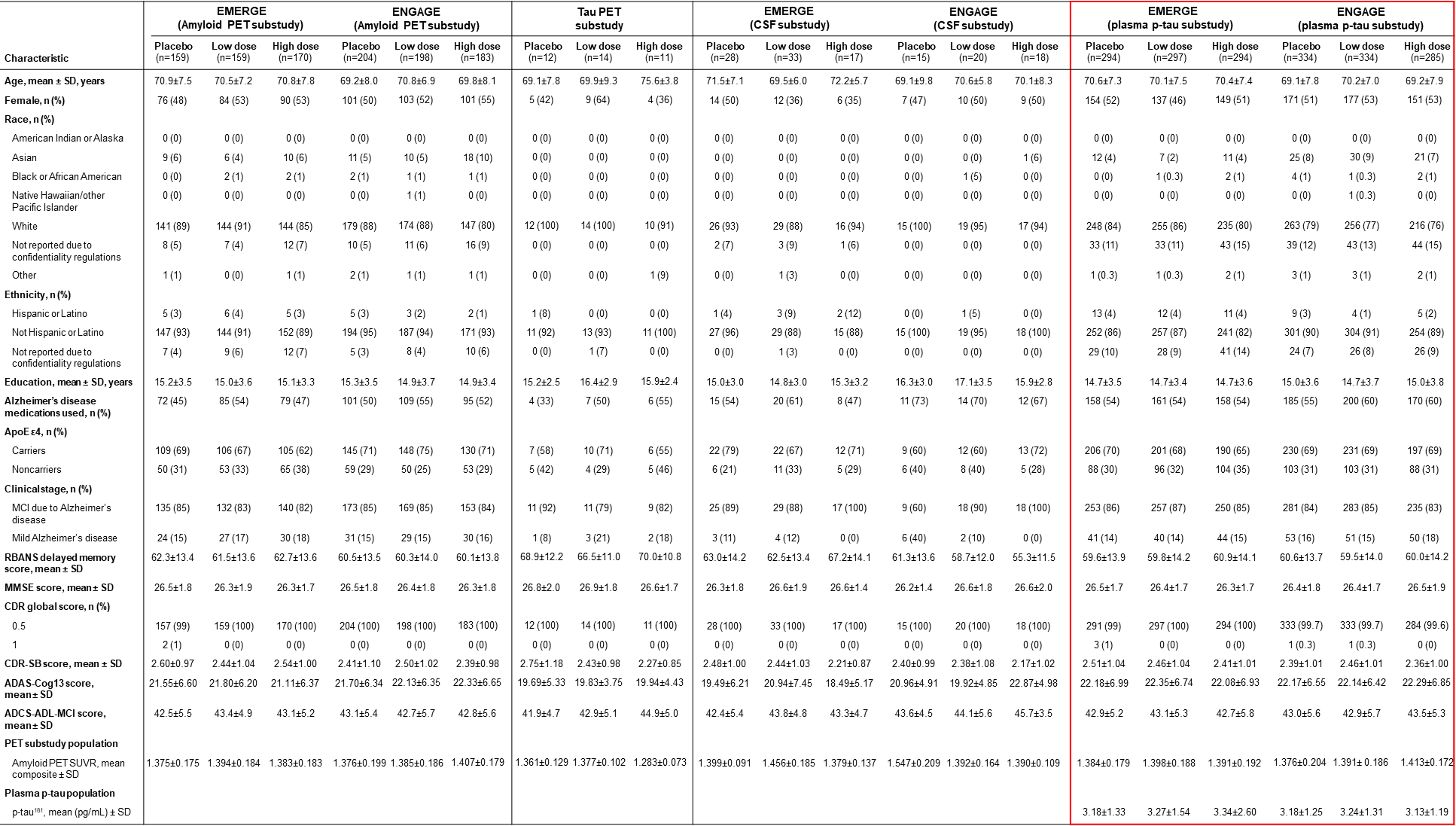
**

Demographics and baseline characteristics are shown for the amyloid PET substudy population, the tau PET substudy population, the CSF substudy population, and the plasma p-tau^181^ analysis population.

ADAS-Cog13, Alzheimer’s Disease Assessment Scale–cognitive subscale (13 items); ADCS-ADL-MCI, Alzheimer’s Disease Cooperative Study–Activities of Daily Living Inventory (mild cognitive impairment version); ApoE, apolipoprotein E; CDR-SB, Clinical Dementia Rating–sum of boxes; MCI, mild cognitive impairment; MMSE, Mini-Mental State Examination; PET, positron emission tomography; RBANS, Repeatable Battery for Assessment of Neuropsychological Status; SUVR, standardized uptake value ratio.

**Supplemental Data Fig. 4**


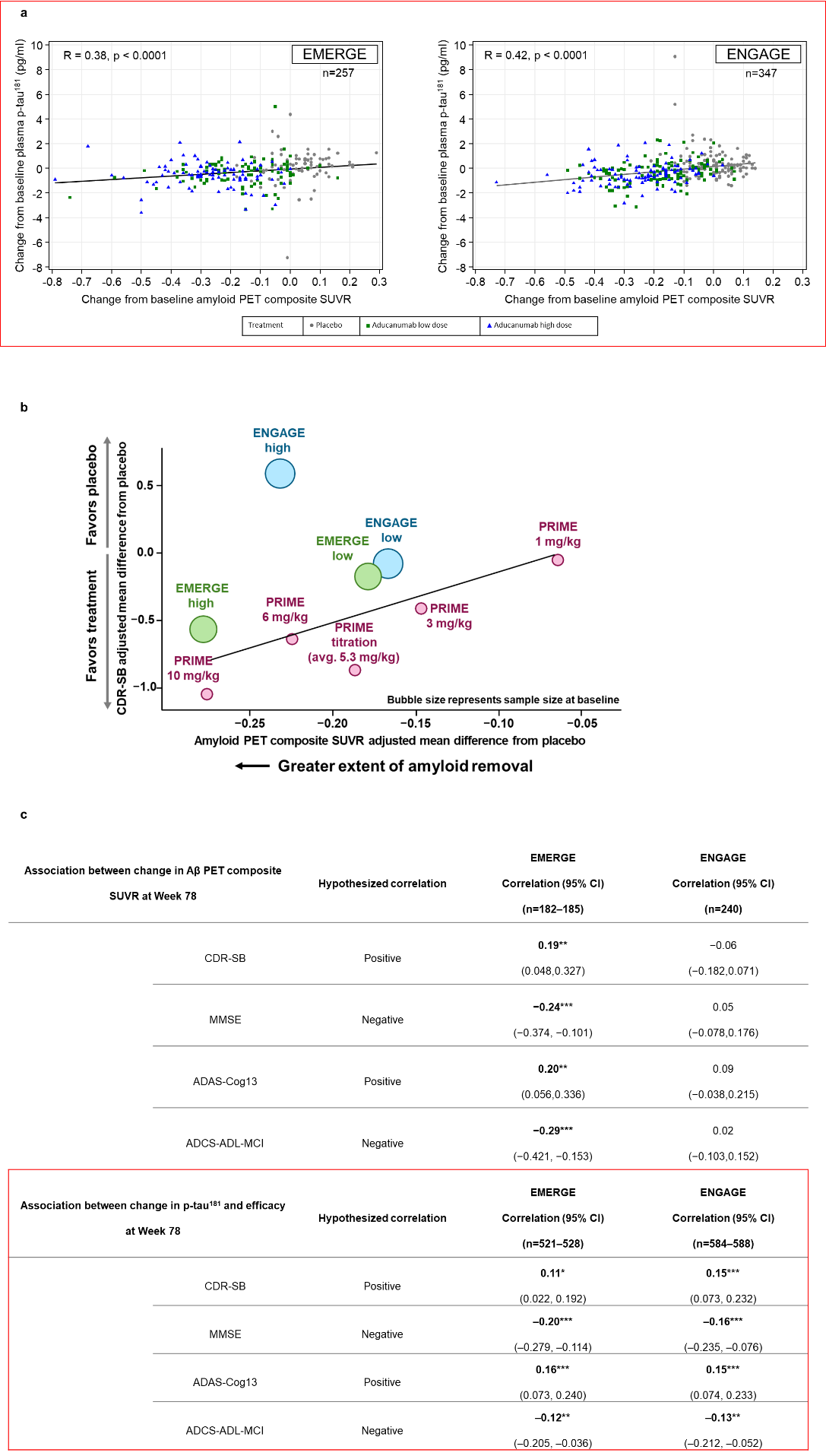


**Panel a** shows a scatterplot of change from baseline in plasma p-tau^181^ levels vs. change from baseline in amyloid PET composite SUVR at Week 78 in EMERGE (left) and ENGAGE (right). R: Partial spearman correlation adjusted for baseline p-tau, baseline amyloid PET, and age. Correlations calculated based on all arms.

**Panel b** shows association between treatment effect on brain Aβ plaque levels and CDR-SB across aducanumab studies (group-level analysis). The analysis was conducted in active treatment groups, as pre-specified. CDR-SB results for EMERGE and ENGAGE were from the PET substudies using the same mixed model for repeated measures as the primary analysis for CDR-SB. The regression line was derived based on the data points from all three studies except the ENGAGE high-dose group. Sample sizes for each study are as follows: EMERGE (n=159 for low dose; n= 170 for high dose); ENGAGE (n=198 for low dose; n=183 for high dose); PRIME (n=29 for 1 mg/kg; n=32 for 3 mg/kg; n=30 for 6 mg/kg; n=31 for 10 mg/kg; n=19 for titration).

**Panel c** shows correlations between amyloid reduction or reduction in levels of plasma p-tau^181^ and efficacy endpoints change from baseline at week 78 (participant-level analysis). The population is limited to those participants in the amyloid PET or plasma p-tau^181^ subgroup who completed amyloid PET assessment or collection of plasma p-tau^181^ and efficacy assessments at week 78. P values (nominal): **P*<.05 ** *P*<.01, *** *P*<.001. Correlations are partial Spearman correlations assessed in pooled low- and high-dose groups after adjustment for baseline biomarker and efficacy values (and age for correlation between plasma p-tau^181^ and efficacy correlation).

Aβ, amyloid β; ADAS-Cog13, Alzheimer’s Disease Assessment Scale–cognitive subscale (13 items); ADCS-ADL-MCI, Alzheimer’s Disease Cooperative Study–Activities of Daily Living Inventory, avg, average; mild cognitive impairment version; CDR-SB, Clinical Dementia Rating Scale–sum of boxes; MMSE, Mini Mental State Examination; PET, positron emission tomography; p-tau, phosphorylated tau; SUVR, standardized uptake value ratio.
